# Fermentation of Sainfoin Seed Flour with *Saccharomyces boulardii*: Effects on Total Dietary Fiber, Anti-Nutrients, Antimicrobial Activity, and Bioaccessibility of Bioactive Compounds

**DOI:** 10.3390/microorganisms13061421

**Published:** 2025-06-18

**Authors:** Havva Polat Kaya, Burcu Kaya, Necati Barış Tuncel, Gulay Ozkan, Esra Capanoglu, Seedhabadee Ganeshan, Mehmet Caglar Tulbek

**Affiliations:** 1 Department of Food Technology, Faculty of Applied Sciences, Çanakkale Onsekiz Mart University, Çanakkale 17100, Türkiye; havva.polat@comu.edu.tr; 2 Department of Food Engineering, Faculty of Engineering, Çanakkale Onsekiz Mart University, Çanakkale 17100, Türkiye; burcu.kaya@comu.edu.tr (B.K.); baristuncel@comu.edu.tr (N.B.T.); 3 Department of Food Engineering, Faculty of Chemical-Metallurgical Engineering, Istanbul Technical University, İstanbul 34469, Türkiye; ozkangula@itu.edu.tr (G.O.); capanogl@itu.edu.tr (E.C.); 4 Saskatchewan Food Industry Development Centre Inc. (Food Centre), Saskatoon, SK S7M 5V1, Canada; pganeshan@foodcentre.sk.ca

**Keywords:** sainfoin seed flour, fermentation, *Saccharomyces boulardii*, anti-nutrients, antioxidants, bioaccessibility

## Abstract

This study investigates the effects of fermentation on sainfoin seed flour using *Saccharomyces boulardii* for total dietary fiber (TDF) content, anti-nutritional profiles (including phytates, tannins, saponins, and trypsin inhibitors), and bioactive compounds. It also focused on assessing the in vitro availability of phenolic compounds, antioxidant potential, and anti-nutrient compounds after gastrointestinal digestion. Four treatment groups were designed: a non-fermented control group, and flour samples fermented with *S. boulardii* CNCM I-745 for 24, 48, and 72 h. All fermentations were carried out at 30 °C. The effects of fermentation and the analysis results were statistically evaluated at the significance level of *p* < 0.05, and significant differences were detected. Fermentation significantly increased soluble dietary fiber (from 3.32% to 4.43%) and reduced anti-nutritional factors, including phytates (by 18%), tannin (by 19%), and trypsin inhibitor activity (TIA) (by 79%). However, saponin content increased by 21% after 72 h of fermentation. Tannin levels of non-fermented and fermented sainfoin flour decreased dramatically after in vitro digestion. Moreover, it was concluded that the bioaccessibility of phytic acid significantly increased through fermentation, while that of tannins declined. Antimicrobial activity against *Escherichia coli* ATCC 25922 improved after fermentation, while the antioxidant capacity was enhanced post-digestion. In addition, the highest phenolic content (612 mg GAE/100 g) and antioxidant capacity (1745 mg TE/100 g by CUPRAC assay and 1127 mg TE/100 g by DPPH assay) were determined in fermented sainfoin seed flour at 72 h after gastrointestinal digestion.

## 1. Introduction

Addressing the growing global demand for food due to increasing population pressures has become a critical challenge. The focus has shifted towards identifying cost-effective, abundant, and sustainable protein sources [1]. In this context, alternative protein sources such as plants, algae, fungi, insects, and microbial proteins are gaining attention [2]. However, the significant environmental impact of animal protein production, coupled with the rising popularity of vegan diets and the limited consumer acceptance of insects as food, has driven a growing interest in plant-based proteins [3]. Consequently, the development of plant-based products as substitutes for animal-derived foods, along with efforts to enhance their nutritional and sensory qualities, has become a priority. Additionally, incorporating underutilized, high-protein plant species into the diet is increasingly recognized for its importance. Sainfoin (*Onobrychis viciifolia*) is a perennial forage legume with considerable potential for plant breeding, thanks to its genetic diversity. Its deep root system supports efficient nutrient absorption, and its high nitrogen-fixing capacity enriches soil fertility without requiring nitrogen fertilizers [4,5]. Beyond its agricultural benefits, sainfoin seed is also a promising plant-based food source for humans due to its high protein content, which has been reported at 42%. It has been reported that sainfoin seeds also contain 45% dietary fiber, 9% crude fat, and significant levels of potassium and magnesium. However, they are particularly high in anti-nutritional substances, especially tannins [6], and would need to be further biotransformed for it to become acceptable for human consumption.

One of the approaches to enhance functionality of sainfoin as a food product is by fermentation. Many bacterial and fungal species have been shown to improve the nutritional profiles of leguminous species including by reducing the anti-nutritional factors of phytic acid, tannins, and trypsin inhibitors [7]. Among these microorganisms, probiotic types have attracted interest due to their beneficial gut health improvement propensities in addition to their abilities to modify flours [8,9]. While lactic acid bacteria (LAB) have been mostly associated with changes in flavor and aroma profiles as well as reduction in anti-nutritional factors in leguminous species during fermentation [10], several probiotic yeast species have also led to similar changes. For example, *Saccharomyces cerevisiae* var. *boulardii* (*S. boulardii*), a probiotic yeast capable of thriving at 37 °C and low pH levels [11,12], produces bioactive compounds such as alcohol, carbon dioxide, gamma-aminobutyric acid (GABA), and B vitamins, including thiamine, riboflavin, biotin, and pyridoxine. It also synthesizes isoflavones and phenolic compounds, such as vanillin, vanillic acid, and cinnamic acid [11,13]. Additionally, *S. boulardii* enhances the nutritional value of foods by synthesizing folates and degrading anti-nutrients such as phytate [14]. Existing studies reveal that fermenting various substrates with *S. boulardii* significantly reduces antinutritional factors, including trypsin inhibitors, phytic acid, alkaloids, glucosinolates, and 3-butyl isothiocyanate [8,15,16]. Moreover, it has been reported that the biochemical profile of the final fermentation products largely depends on the fermentation duration [16]. The aim was to evaluate the impact of varying fermentation times (24, 48, and 72 h) on the dietary fiber content, levels of anti-nutrients, antimicrobial activity, and the bioaccessibility of both anti-nutrients and bioactive compounds in sainfoin seed flour following in vitro digestion.

## 2. Materials and Methods

### 2.1. Substrate and Strain Preparation for Fermentation

Sainfoin seeds were obtained from local markets and were dehulled using a laboratory-scale rice milling system (CRM-1252T, Yaşar Makina, Samsun, Türkiye). Andaç et al. [6] reported on the proximate composition of sainfoin seeds as consisting of 45.24% TDF, 42.21% crude protein, 8.91% crude fat, and 4% ash. The sainfoin used in this study is the same as that used in the study by Andaç et al. *S. boulardii* CNCM I-745 was purchased from Biocodex as lyophilized stock (California, CA, USA). The pure culture was revived by incubating it in yeast extract peptone broth, which consisted of 10 g/L yeast extract (Biolife, Milan, Italy), 20 g/L peptone (Oxoid, Basingstoke, UK), and 20 g/L glucose (Carlo Erba, Milan, Italy), pH adjusted to 6.5 at 30 °C for 48 h. The reconstituted yeast strain was maintained on PDA (Potato Dextrose Agar) plates. For fermentation, 25 g of sainfoin flour was mixed with 50 mL of sterile water and inoculated with *S. boulardii* at a concentration of 1.5 × 10^4^ CFU/g. A control was also set up and consisted only of 25 g sainfoin flour mixed with 50 mL sterile water without inoculation with *S. boulardii*. In preliminary studies no effects of indigenous microorganisms were observed. Fermentation was conducted at 30 °C with 50 rpm (Jeio Tech/IS-97, Seul, Republic of Korea) for three different durations: 24, 48, and 72 h. The fermented samples were subjected to freeze-drying at under 0.0033 mbar vacuum at −90.6 °C for 24 h prior to analysis.

### 2.2. Growth Dynamics During Fermentation

The viable cell counts over the duration of the fermentation were determined using the serial dilutions. Ten grams of the fermented sample was mixed with 90 mL of physiological saline (0.85% NaCl, *w*/*v*) to prepare a 10^−1^ dilution. Serial dilutions were then created from this initial mixture. From the prepared serial dilutions, 100 µL samples were plated onto a PDA (Oxoid, UK) medium using the spread plate method. The plates were incubated at 30 °C for 48 h, after which colony counts were performed. Subsequently, the viable *S. boulardii* count in the fermented samples was expressed as CFU/g [17].

### 2.3. Total Dietary Fiber (TDF) Content Determination

The TDF amounts of fermented and non-fermented sainfoin seed flour were determined using the Megazyme Total Dietary Fiber Assay Kit (Megazyme Wicklow, Wicklow, Ireland) according to the enzymatic-gravimetric method (No: 32-07) specified in the AACC standard [18].

### 2.4. Anti-Nutrients

#### 2.4.1. Phytic Acid Determination

Phytic acid content was determined according to Gao et al. [19] using the Wade reagent method. Briefly, samples (0.5 g) were weighed into 15 mL centrifuge tubes. To each, 10 mL of 2.4% (0.64 N) HCl was added and vortexed for 10 s. Tubes were shaken at 300 rpm for 16 h at room temperature, then centrifuged at 8500× *g*, 10 °C for 20 min. The supernatant was filtered into tubes containing 1.0 g NaCl and vortexed to dissolve the salt. The mixture was shaken at 300 rpm for 20 min and allowed to settle at 4 °C for 60 min. Following a second centrifugation at 3000 rpm and 10 °C for 20 min, 1 mL of supernatant was diluted to 25 mL with deionized water. From this, 3 mL was transferred into a tube and mixed with 1 mL Wade reagent (30 mg of FeCl_3_.6H_2_O and 300 mg of sulfosalicylic acid in 100 mL distilled water). After vortexing and centrifugation at 3000 rpm and 10 °C for 10 min, absorbance was measured at a 500 nm wavelength in a spectrophotometer (Shimadzu/UV-160A, Kyoto, Japan) against a distilled water blank. To generate a standard curve, sodium phytate solution was used.

#### 2.4.2. Saponin Determination

The saponin content of samples was analyzed using a spectrophotometric method, according to a previously published report [20], with some modifications. For extraction, 1 g of sainfoin seed flour and 5 mL of methanol/water (80:20, *v*/*v*) were mixed in an orbital shaker at 150 rpm and 25 °C overnight. The suspension was centrifuged at 4000× *g* for 5 min at 4 °C, and this procedure was repeated twice. Then, 0.2 mL of supernatant, 0.3 mL of methanol/water (80:20, *v*/*v*), 0.5 mL of 8% vanillin (in methanol and water (50:50, *v*/*v*), and 5 mL of sulfuric acid (72%) were mixed and incubated at 60 °C for exactly 10 min. The absorbance of the mixture was measured at 544 nm against methanol/water (80:20, *v*/*v*) and the results were expressed as soyasaponin I equivalents.

#### 2.4.3. Tannin Determination

Tannin determination was conducted according to a previously published report [20], with some modifications. Briefly, tannins were extracted with 20 mL water from non-fermented and fermented seed flour (0.2 g) by continuous vortexing for 2 min. After centrifugation (8000× *g*, 5 min, 25 °C) and filtration, 4 mL of tannin extract was transferred to a test tube and mixed with 2 mL of water/methanol (1:1, *v*:*v*) and 1 mL of 4-(Dimethylamino)-cinnamaldehyde (DMACA) solution (Millipore-Sigma, Burlington, MA, USA, Cat # 49825-50ML-F). The mixture was incubated at room temperature for 30 min and absorbance was measured at 640 nm against a water/methanol (1:1, *v*:*v*) mixture. Tannin contents of the samples were expressed as catechin equivalents.

#### 2.4.4. Determination of Trypsin Inhibitor Activity (TIA)

TIA was determined according to the official method of AACC Method # 22-40 [18]. Briefly, non-fermented and fermented sainfoin seed flours were extracted with 0.01 N NaOH for 3 h, and diluted suspensions were treated with *N*_α_-Benzoyl-L-arginine 4-nitroanilide hydrochloride (BAPA) (Sigma, Burlington, MA, USA, Cat# B3279-100MG) as substrate at 37 °C for 10 min in the presence of trypsin enzyme (salt-free, ≥10,000 BAAE units/g protein). The reaction was stopped by adding 1 mL of acetic acid solution (30%, *v*/*v*) after exactly 10 min. One trypsin unit is arbitrarily defined as the increase of 0.01 absorbance units at a 410 nm wavelength per 10 mL of reaction mixture. Trypsin inhibitor activity is expressed in terms of trypsin inhibitor units (TIU) per mg sample.

### 2.5. Antimicrobial Activity

*Staphylococcus aureus* ATCC 6538, *Bacillus cereus* ATCC 11778, *Escherichia coli* ATCC 25922, and *Klebsiella quasipneumoniae* ATCC 700603 were tested to determine the antimicrobial activity of the non-fermented and fermented sainfoin seed flour samples. The extraction of antimicrobial compounds was performed according to Miedzianka et al. [21], with some modifications. Two grams of the fermented sample were mixed with 10 mL of 75% methanol, and then centrifuged at 5500× *g* for 5 min. The extracts were sterilized using a 0.22 µm syringe filter.

#### 2.5.1. Minimum Inhibitory Concentration (MIC) and Minimum Bactericidal Concentration (MBC)

Serial two-fold dilutions of sample extracts in concentrations ranging from 200 mg/mL to 3.12 mg/mL with adjusted bacterial concentrations (10^5^ CFU/mL, 0.5 McFarland standard) were used to determine the minimum inhibitory concentration in Mueller Hinton broth (Merck, Darmstadt, Germany). Each tube was incubated at 37 °C for 24 h. Bacterial growth was determined based on the formation of a pink-red color after the addition of 100 µL of 1% tetrazolium to each tube and incubation at room temperature for 10–15 min. In the well containing the lowest concentration of the sample extract that inhibited bacterial growth, no pink-red coloration was observed, and the preceding well represented the MIC [22]. Two tubes showing no growth, along with the tube corresponding to the MIC value, were spot inoculated with 10 µL onto Mueller Hinton agar. The concentration at which no growth was observed after 24 h of fermentation at 37 °C was determined as the MBC.

#### 2.5.2. Agar Well Method

The microorganisms were incubated in Tryptic Soy Broth (Merck, Darmstadt, Germany) at 37 °C and adjusted to 0.5 McFarland. A 100 mL bacterial culture was spread over the surface of Mueller-Hinton agar (Oxoid, UK). Then, 10 µL of the sample extract (100 mg/mL) was added to wells with a 6.50 mm diameter. After incubation at 37 °C for 24 h, the diameters of the inhibition zones on the plates were measured. For each microorganism, all fermented samples were tested in triplicate [23].

### 2.6. Extraction Procedure of Phenolic Compounds

Extractions of phenolic compounds from non-fermented and fermented sainfoin seed flours were performed as reported previously [24]. Briefly, 0.5 g of each sample was mixed with 5 mL of 75% methanol (*v*/*v*). This mixture was sonicated for 15 min using an ultrasonic bath (USC900TH; VWR, Radnor, PA, USA) at ambient temperature, and subsequently centrifuged (Universal 32R; HettichZentrifugen, Tuttlingen, Germany) at 4000 rpm (2700× *g*) and 4 °C for 10 min. Then, the supernatant was separated from filtrate. This procedure was repeated one more time using the remaining filtrate, supernatants were pooled, and the volume was adjusted to 10 mL with 75% methanol. The extracts were stored at −20 °C for further analyses.

### 2.7. In Vitro Gastrointestinal Digestion Simulation

Non-fermented and fermented sainfoin seed flours were subjected to a standardized static in vitro gastrointestinal digestion model with some modifications [25]. For the oral digestion, 2.5 g of each sample was mixed with 4 mL of salivary fluid and 25 μL of CaCl_2_ (0.3 M). The total volume was adjusted to 10 mL with distilled water, and this mixture was incubated in a water bath at 37 °C for 2 min with shaking. In the gastric phase, 7.5 mL of gastric fluid and 5 μL of CaCl_2_ (0.3 M) were added to each sample, the pH was adjusted to 3 using 1 M HCl, and the mixture was mixed with 1.6 mL of pepsin (25,000 U/mL). The total volume was adjusted to 20 mL with distilled water, and this mixture was incubated in a water bath (Memmert SV 1422, Nürnberg, Germany) at 37 °C for 2 h with shaking. After incubation, 5 mL of aliquots were separated from each sample for further examination. In the intestinal phase, 8.25 mL of intestinal fluid and 30 μL CaCl_2_ (0.3 M) were added to the remaining samples, the pH was adjusted to 7 by using 1 M NaOH, and then the mixture was mixed with 3.75 mL of pancreatin (800 U/mL) and 1.875 mL of bile (160 mmol/L). The total volume was adjusted to 30 mL with distilled water, and this mixture was then incubated in a water bath at 37 °C for 2 h with shaking. Samples collected from gastric and intestinal phases were immediately placed in ice water and followed by centrifugation (Hettich Zentrifugen Universal 32R, Tuttlingen, Germany) at 14,000× *g* for 30 min to separate bioaccessible and residual fractions from each other. The resulting supernatants were stored at −20 °C for subsequent analysis.

Bioaccessibility of phenolic compounds, antioxidant capacity, tannin, and phytic acid were calculated according to the following equation:

$$\begin{array}{l} Bioaccessibility\;\left(\%\right)\\ =\frac{The\;amount\;of\;total\;phenolics,\;antioxidant\;capacity,\;tannin,\;or\;phytic\;acid\;after\;gastric\;or\;gastrointestinal\;digestion}{The\;amount\;of\;total\;phenolics,\;antioxidant\;capacity,\;tannin,\;or\;phytic\;acid\;before\;digestion\;}\times 100 \end{array}$$

A blank sample (containing the same amount of water instead of non-fermented and fermented sainfoin seed flours) was also incubated under the same digestion conditions and used to avoid any interferences arising from the digestive fluids.

### 2.8. Determination of Total Phenolic Content

Total phenolic content (TPC) of the extracts and samples collected from gastric and intestinal digestion steps were determined according to Singleton and Rossi [26]. The TPC of the samples from before and after digestion was calculated using a calibration curve, and the results were stated as mg of gallic acid equivalent (GAE) per 100 g sample.

### 2.9. Determination of Total Antioxidant Capacity

Total antioxidant capacity (TAC) of the extracts and samples collected from gastric and intestinal digestion steps were calculated by applying the copper (II) ion reducing antioxidant capacity (CUPRAC) [27] and the (1,1-diphenyl-2-picrylhydrazil) (DPPH) [28] assays. The TAC of the undigested and digested samples was determined using a calibration curve, and the results were shown as mg Trolox equivalents (TE) per 100 g sample.

### 2.10. Statistical Analyses

The objective was to assess how different fermentation durations (24, 48, and 72 h) affect the dietary fiber, anti-nutrient levels, and antimicrobial activity as well as the bioaccessibility of anti-nutrients and bioactive compounds in sainfoin seed flour after in vitro digestion. The effect of the fermentation period on the above-mentioned properties was assessed with One-Way Analysis of Variance (ANOVA) at a significance level of 95% (*p* < 0.05) using MINITAB (ver. 20, StatSoft, Inc., Tulsa, OK, USA) and SPSS (version 22.0, SPSS, Chicago, IL, USA) software. Multiple comparisons were performed by Tukey’s test. All results were presented as mean (at least three replicates for all analyses) ± standard error.

## 3. Results

### 3.1. Viable Cell Count in Fermented Sainfoin

**Figure 1:**
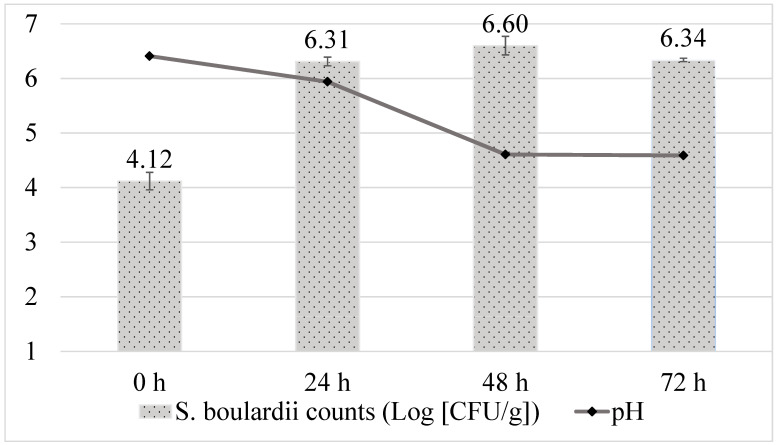
Changes in cell counts of *S. boulardii* and pH in fermented sainfoin flour over time.

The viable cell counts of *S. boulardii* increased by 2 log units following fermentation (Figure 1). The increase in the CFU with fermentation was significant (*p* < 0.05). Additionality *S. boulardii* maintained viability and viable cell counts for 72 h on sainfoin seed flour. The CFU of viable cell counts with different fermentation times was not significant (*p* > 0.05).

### 3.2. Total Dietary Fiber Content

In both fermented and non-fermented sainfoin flours, the predominant fiber fraction was the insoluble dietary fiber (IDF) (Table 1). IDF showed a slight increase from 30.82% in non-fermented samples to a maximum of 33.06% after 48 h of fermentation, although this change was not statistically significant (*p* > 0.05). In contrast, soluble dietary fiber (SDF) increased significantly (*p* < 0.05) across all fermentation durations, peaking at 4.43% after 72 h. TDF also increased significantly (*p* < 0.05), from 33.32% in non-fermented samples to 37.26% after 72 h of fermentation. The increase in SDF content contributed to the increase in TDF.

### 3.3. Anti-Nutrient Compounds

The anti-nutritional compound composition of non-fermented sainfoin seed flour includes phytic acid at 2.50 mg/g, saponin at 0.05 mg/g, tannin at 77.81 mg catechin equivalents per gram, and trypsin inhibitory activity (TIA) at 3.77 TIU/mg. The fermentation process significantly (*p* < 0.05) affected the anti-nutrient composition of sainfoin seed flour, as shown in Table 2. This effect is evident with changes in the levels of phytate, saponin, tannin, and TIA. Phytate content decreased significantly (*p* < 0.05) throughout the fermentation process from 2.50 mg/g in the non-fermented sample to 2.05 mg/g after 72 h of fermentation, showing an 18% reduction.

Sainfoin seed flour, known for its high tannin content, was found to contain 77.81 mg/g of tannins. The tannin content gradually increased significantly with the extension of the fermentation period, peaking at a 19% increase after 72 h (*p* < 0.05). Trypsin inhibitor activity (TIA) significantly decreased (*p* < 0.05) during fermentation, dropping from 3.77 TIU/mg in non-fermented samples to 0.78 TIU/mg after 72 h. Saponin content, however, showed a significant increase (*p* < 0.05), increasing from 0.05 mg/g in non-fermented samples to 1.10 mg/g after 72 h of fermentation. In summary, fermentation led to a reduction in anti-nutritional factors such as phytate, tannin, and TIA, while increasing saponin levels. These changes collectively enhance the nutritional and functional quality of sainfoin seed flour, making it a promising ingredient for the development of health-promoting food products.

### 3.4. Antimicrobial Activity

The antimicrobial activity of fermented sainfoin seed flour at 24 h, 48 h, and 72 h of fermentation is shown in Table 3. The fermented and non-fermented sainfoin exhibited inhibition activity against all tested microorganisms. The MIC values of non-fermented and fermented sainfoin seed flour against *Staphylococcus aureus* ATCC 6538 (25 mg/mL), *Bacillus cereus* ATCC 11778 (12.5 mg/mL), and *Klebsiella quasipneumoniae* ATCC 700603 (12.5 mg/mL) were constant. However, the MIC value for *Escherichia coli* ATCC 25922 decreased from 50 mg/mL to 25 mg/mL (by 50%), indicating increased susceptibility with 24 h fermentation. The MBCs of all samples were constant for *Bacillus cereus* ATCC 11778 (25 mg/mL), *Escherichia coli* ATCC 25922 (50 mg/mL) and *Klebsiella quasipneumoniae* ATCC 700603 (25 mg/mL). 

### 3.5. Effects of In Vitro Gastrointestinal Digestion on the Content of Anti-Nutritional Compounds

The in vitro digestion assay results for the anti-nutritional factors (phytate and tannin) in non-fermented and fermented sainfoin flour after gastric and intestinal digestion are summarized in Table 4. The data show significant changes (*p* < 0.05) in both the bioaccessibility and degradation of phytate and tannins during the digestive process as influenced by fermentation.

Phytate content exhibited a notable decrease during digestion, with the undigested (UD) phytate content in non-fermented sainfoin flour measured at 2.50 mg/g. After gastric digestion (GD), it decreased to 0.44 mg/g (by 82.4%), and following intestinal digestion (ID), it increased slightly to 1.61 mg/g. This trend was consistent among samples fermented for 24 h, 48 h, and 72 h. Unlike undigested samples, during gastric digestion, the amount of phytic acid significantly increased with the fermentation period. To the best of our knowledge, no data regarding changes in phytic acid content after in vitro digestion have been reported in the literature. Tannin content followed a different pattern, with undigested levels in non-fermented samples recorded at 77.81 mg/g. After gastric digestion, tannin content decreased to 8.89 mg/g (by 88.55%) and further declined to 5.24 mg/g (93.28%) after intestinal digestion. In both non-fermented and fermented samples, tannin content gradually decreased at each stage of in vitro digestion. The bioaccessibility of phytate increased after fermentation, particularly after 72 h of fermentation, reaching 80.22%. In contrast, the bioaccessibility of tannins significantly decreased as the fermentation period was extended. In non-fermented samples, tannin bioaccessibility was 6.74%, which declined to 3.82% in samples fermented for 72 h.

### 3.6. Effects of In Vitro Gastrointestinal Digestion on the Content of Phenolic Compounds and Antioxidant Capacity

The total phenolic content of the non-fermented and fermented sainfoin seed flour samples was significantly enhanced after gastrointestinal digestion when compared to that of undigested sainfoin seed flour (*p* < 0.05). While there was an increase after gastric digestion, in general, phenolic content declined after the intestinal digestion phase except for in the 0 and 72 h fermented flour samples (Table 5). Despite the fact that the bioaccessibility (%) value was remarkably reduced by fermentation from 225% to 100–181%, the actual phenolic content of the samples still showed an increasing trend by fermentation, especially from 48 h (566 mg GAE/100 g).

Similar to the outcomes of total phenolic content results, there was a noticeable increase in the antioxidant capacity of non-fermented and fermented sainfoin seed flour samples after gastrointestinal digestion, as measured by DPPH assay, compared to the undigested samples. However, concerning the antioxidant capacity of the samples analyzed by CUPRAC assay, the bioaccessibility (%) value was remarkably reduced by fermentation, yet the actual value (1745 mg TE/100 g) reached a maximum at 72 h of fermentation. Thus, the digested sainfoin seed flour products with different fermentation times exhibited various antioxidant potentials.

## 4. Discussion

The increase in CFU and 3-day stability in viability were consistent with the findings of Ryan et al. [29] in rice bran as a medium. Minerals such as potassium and magnesium play essential roles in the metabolism of yeast cells and ATP synthesis [30]. Andaç et al. [6] reported the presence of minerals such as Zn, Se, Mg, K, Ca, Fe, and Cu. By comparing the findings of this study with those in the literature, it can be concluded that sainfoin serves as an adequate substrate for the growth of *S. boulardii*. The pH value of sainfoin seed flour decreased from 6.41 to 4.59 following fermentation. This can be associated with *S. boulardii* utilizing the sugar in the substrate to produce organic acids [8].

Similar results for SDF and TDF were obtained from the solid-state fermentation of wheat flour with *Lactobacillus plantarum* [31]. This outcome may be attributed to the synthesis of non-cellular soluble fiber structures, such as β-glucan, during fermentation [32]. Soluble fibers have been linked to improved gut health, reduced cholesterol levels, and better glycemic control [33].

The anti-nutrient profile observed in this study is consistent with the findings of Andaç et al. [6], as the decrease in phytic acid content is indirectly linked to an increase in phytase enzyme activity, stimulated by the reduction in pH during fermentation [8]. Phytase production by *Saccharomyces* spp. has been extensively reported [34], and *S. boulardii* has also been shown to hydrolyze phytate [35]. This is due to the action of repressible acid phosphatases secreted extracellularly [36]. Indeed, fermentation is an effective method for reducing phytic acid content in various grains and legumes, as also demonstrated by *Rhizopus oligosporus* and bakery yeast in prior studies [37,38].

In a manner similar to the results of the present study, reductions in tannin content were observed [6,39,40]. In the literature, fermentation using lactic acid bacteria has been shown to effectively reduce tannin levels in various plant-based materials. *Lactobacillus plantarum* was particularly effective at reducing tannin content in sorghum [41], while a combination of *Saccharomyces cerevisiae* and *Acetobacter aceti* proved successful in cabbage fermentation [42]. The reduction in tannins is primarily attributed to the activity of tannase enzymes produced by microorganisms [42]. Tannase activity of yeast was reported by Aoki et al. [43]. Nivetha et al. [44] reported that the tannin content in the production of flaxseed beverage was reduced by 33% following *S. boulardii* fermentation.

The reduction of TIA may be related to the increased microbial protease activity during fermentation process [45]. Moré and Vandenplas [46] reported that *S. boulardii* CNCM I-745 increases protein and peptide hydrolysis through multiple mechanisms. Furthermore, Castagliuolo et al. [47] demonstrated that *S. boulardii* secretes a 54-kDa protease that inhibits *Clostridium difficile* toxins. Sourdough fermentation has been shown to effectively degrade wheat α-amylase/trypsin inhibitors, thereby reducing their inflammatory activity [48]. In legumes such as soybean, lentil, and green pea, fermentation with lactic acid bacteria, especially when combined with physical processing techniques like sonication or pre-cooking, resulted in a substantial decline in TIA [49]. Furthermore, this is consistent with recent studies exploring the impact of fermentation on saponin content in various plant materials. Toor et al. [50] reported similar findings, noting that fermentation with *Rhizopus oligosporus* for 52 h resulted in a 40% increase in saponin content in fermented kabuli chickpeas, 16% in desi chickpeas, and 28% in pigeon peas, while a 12% decrease was observed in soybeans. Additionally, lactic acid fermentation of soy saponins using *Lactobacillus rhamnosus* altered the saponin profile by reducing 2,3-dihydro-2,5-dihydroxy-6-methyl-4H-pyran-4-one (DDMP)-conjugated saponins and increasing soyasaponins I and III [51]. This change was attributed to the synthesis of saponins as a defense mechanism during fermentation. While saponins are generally reported as anti-nutritional factors, they have also been noted for their bioactive properties due to their antioxidant effects [52].

Butkutė et al. [53] determined that the ethanol extract of sainfoin seed exhibits antimicrobial activity against *Staphylococcus aureus* ATCC 25923, *Staphylococcus epidermidis* ATCC 12228, *Enterococcus faecalis* ATCC 29212, *Bacillus cereus* ATCC 11778, *Bacillus subtilis* ATCC 6633, *Klebsiella pneumoniae* ATCC 13883, and *Pseudomonas aeruginosa* ATCC 27853. Furthermore, researchers found that *E. coli* ATCC 2922 was not susceptible to the extracts in the concentration of 50 mg/mL. Regarding antimicrobial activity, the results of this study are similar to that of Liu et al. [54] who reported the MIC value against different strains of *E. coli* and *E. coli* O157:H7 for sainfoin condensed tannins to be in the range of 100 to 150 µg/mL. The reason for lower antimicrobial activity detected in this study may be related to the product not being a condensed fraction. In contrast to MIC and MBC results, no inhibition zone was observed in test microorganisms (except *Bacillus cereus* ATCC 11778) according to the agar well method. This can be explained by the fact that these strains were not susceptible to the extract at a concentration of 100 mg/mL.

After in vitro digestion, fermentation markedly reduced anti-nutritional compounds and improved both the bioaccessibility of phenolic substances and antioxidant properties in sainfoin seed flour. The low pH (e.g., the stomach pH) may have caused phytic acid to form insoluble complexes with protein residues through electrostatic interactions, thereby reducing its solubility [55]. It has been reported that during the fermentation of polyphenol-rich food products, tannin as a phenolic substance with a high degree of polymerization could be hydrolyzed into smaller compounds by the rupture of lipid, glycosidic, and ether bonds by the action of tannase as well as by means of beneficial microorganisms such as yeast, lactic acid bacteria, and *Bifidobacterium* [56].

Mencin et al. [57] indicated that the total phenolic content of spelt seeds (*Triticum spelta* L. cv. *Ostro*) fermented for 72 h at 30 °C improved after digestion, in agreement with the results of our study, specifically as the TPC of germinated and fermented seeds increased by 103% compared to raw seeds. The substantial increase in the available fraction of the phenolic compounds in fermented foods after digestion could be related to the hydrolysis of fiber polymers and thus the disruption of cell walls by hydrolytic enzymes [58]. On the other hand, lower phenolic content of the non-fermented samples may be due to conjugation of phenolics to other compounds, such as carbohydrates and dietary fibers, resulting in resistance to digestion [57]. In addition, Sánchez-Velázquez et al. [59] also observed an increasing number of phenolic substances by digestion, which may have an impact on their bioactivity. In parallel with the antioxidant activity results of the present study, Zhou et al. [60] found an increase in the antioxidant capacity of fermented chickpea flour evaluated by ABTS and DPPH methods after intestinal digestion. The increment in the release of the phenolic and flavonoid metabolites as well as bioactive peptide contents may contribute to the ABTS and DPPH radical scavenging abilities of the fermented samples, rather than non-fermented chickpea flour.

## 5. Conclusions

The increase in viable cell counts during fermentation suggests that sainfoin provides a suitable substrate for the growth of *S. boulardii*, and the stability of the cells for up to 72 h further highlights its potential as a probiotic carrier. The fermentation process also led to significant changes in dietary fiber content which could enhance gut health and have other health benefits. Furthermore, the fermentation process notably reduced the content of anti-nutritional compounds while increasing the bioavailability of phytic acid. Additionally, the antimicrobial activity of fermented sainfoin flour remained consistent, with a reduction in the minimal inhibitory concentration (MIC) for *Escherichia coli* after fermentation. Moreover, the fermentation process remarkably reduced the content of anti-nutritional compounds while improving the availability of phenolic substances and antioxidant activity in sainfoin seed flour after digestion. The results indicate that fermented sainfoin seed flour can be a promising ingredient for the development of health-promoting food products, combining improved nutritional quality and enhanced bioaccessibility. The findings suggest that fermentation, especially with *S. boulardii*, can optimize sainfoin as a functional food ingredient.

## Figures and Tables

**Table 1:** Change in dietary fiber content of fermented sainfoin seed flour.

Samples	Insoluble Dietary Fiber (%)	Soluble Dietary Fiber (%)	Total Dietary Fiber (%)
Non-fermented	30.82 ± 0.08	2.51 ± 0.27 ^B^	33.32 ± 0.19 ^B^
0 h fermented	31.10 ± 0.91	5.12 ± 0.34 ^A^	36.23 ± 0.57 ^A^
24 h fermented	33.02 ± 0.88	3.26 ± 0.24 ^B^	36.28 ± 0.64 ^A^
48 h fermented	33.06 ± 0.73	3.28 ± 0.04 ^B^	36.33 ± 0.69 ^A^
72 h fermented	32.83 ± 0.19	4.43 ± 0.02 ^A^	37.26 ± 0.21 ^A^

Within each column, results followed by capital letters are significantly different (*p* < 0.05).

**Table 2:** Anti-nutrient compositions of non-fermented and fermented sainfoin seed flour.

Samples	Phytate (mg/g)	Tannin (mg/g)	TIA (TIU/mg)	Saponin (mg/g)
Non-fermented	2.50 ± 0.00 ^A^	77.81 ± 0.83 ^A^	3.77 ± 0.09 ^A^	0.05 ± 0.01 ^D^
0 h fermented	2.34 ± 0.01 ^B^	73.06 ± 0.25 ^B^	3.29 ± 0.34 ^A^	0.15 ± 0.01 ^D^
24 h fermented	2.21 ± 0.03 ^C^	66.81 ± 0.34 ^C^	1.90 ± 0.01 ^B^	0.35 ± 0.04 ^C^
48 h fermented	2.18 ± 0.02 ^C^	65.12 ± 0.12 ^C^	1.53 ± 0.05 ^B^	0.98 ± 0.01 ^B^
72 h fermented	2.05 ± 0.00 ^D^	62.58 ± 0.53 ^D^	0.78 ± 0.04 ^C^	1.10 ± 0.03 ^A^

TIA: Trypsin inhibitor activity; TIU: Trypsin inhibitor unit. Within each column, results followed by capital letters are significantly different (*p* < 0.05).

**Table 3:** Antimicrobial activity of fermented sainfoin seed flour.

M	MIC (mg/mL)				MBC (mg/mL)				Inhibition Zone (mm) Extract (100 mg/mL)			
	C	24	48	72	K	24	48	72	C	24	48	72
**Sc**	25	25	25	25	50	25	25	50	nd	nd	nd	nd
**Bc**	12.5	12.5	12.5	12.5	25	25	25	25	8.61 ± 0.45	7.43 ± 0.49	7.37 ± 0.15	nd
**Ec**	50	25	25	25	50	50	50	50	nd	nd	nd	nd
**Kq**	12.5	12.5	12.5	12.5	25	25	25	25	nd	nd	nd	nd

M: Microorganisms; C: Control; Sc: *Staphylococcus aureus* ATCC 6538; Bc: *Bacillus cereus* ATCC 11778; Ec: *Escherichia coli* ATCC 25922; Kq: *Klebsiella quasipneumoniae* ATCC 700603; nd: not determined, MIC: minimum inhibitory; MBC: minimum bactericidal concentration.

**Table 4:** Change of antinutritional factors in non-fermented and fermented sainfoin flour after in vitro digestion.

Assays	Samples	UD	GD	ID	Bioaccessibility (%)
Phytate (mg/g)	Non-fermented	2.50 ± 0.00 ^Aa^	0.44 ± 0.01 ^Bb^	1.61 ± 0.01 ^Ac^	64.52 ± 0.17 ^C^
	0 h fermented	2.34 ± 0.01 ^Ba^	0.45 ± 0.01 ^Bc^	1.65 ± 0.02 ^Ab^	70.79 ± 0.83 ^B^
	24 h fermented	2.21 ± 0.03 ^Ca^	0.48 ± 0.02 ^Bc^	1.62 ± 0.01 ^Ab^	73.49 ± 0.13 ^B^
	48 h fermented	2.18 ± 0.02 ^Ca^	0.43 ± 0.03 ^Bc^	1.37 ± 0.02 ^Bb^	62.89 ± 0.95 ^C^
	72 h fermented	2.05 ± 0.00 ^Da^	0.58 ± 0.01 ^Ac^	1.65 ± 0.01 ^Ab^	80.22 ± 0.34 ^A^
Tannin (mg/g)	Non-fermented	77.81 ± 0.83 ^Aa^	8.89 ± 0.02 ^Ab^	5.24 ± 0.09 ^Ac^	6.74 ± 0.11 ^A^
	0 h fermented	73.06 ± 0.25 ^Ba^	8.38 ± 0.08 ^Bb^	4.16 ± 0.13 ^Bc^	5.70 ± 0.18 ^AB^
	24 h fermented	66.81 ± 0.34 ^Ca^	8.95 ± 0.02 ^Ab^	3.63 ± 0.17 ^Bc^	5.44 ± 0.25 ^B^
	48 h fermented	65.12 ± 0.12 ^Ca^	8.84 ± 0.04 ^Ab^	3.41 ± 0.17 ^Bc^	5.23 ± 0.26 ^B^
	72 h fermented	62.58 ± 0.53 ^Da^	6.43 ± 0.05 ^Cb^	2.39 ± 0.24 ^Cc^	3.82 ± 0.39 ^C^

UD: undigested; GD, gastric digestion; and ID: intestinal digestion. Within each column, results followed by capital letters are significantly different (*p* < 0.05). Within each row, results followed by lowercase letters are significantly different (*p* < 0.05).

**Table 5:** Effect of in vitro digestion on phenolic compounds and antioxidant capacity of fermented sainfoin seed flour.

Assays	Period (h)	UD	GD	ID	Bioaccessibility (%)
TPC (mg GAE/100 g	Non-fermented	184 ± 6 ^dC^	526 ± 48 ^bA^	416 ± 48 ^cB^	225 ± 26 ^a^
	0 h fermented	271 ± 27 ^cC^	431 ± 43 ^cB^	491 ± 50 ^bA^	181 ± 18 ^b^
	24 h fermented	335 ± 9 ^bB^	631 ± 68 ^aA^	336 ± 37 ^dB^	100 ± 11 ^d^
	48 h fermented	392 ± 26 ^aB^	603 ± 42 ^abA^	566 ± 59 ^aA^	144 ± 15 ^c^
	72 h fermented	423 ± 16 ^aC^	532 ± 72 ^bB^	621 ± 22 ^aA^	146 ± 5 ^c^
CUPRAC (mg TE/100 g)	Non-fermented	956 ± 79 ^dC^	2035 ± 153 ^bA^	1304 ± 115 ^cB^	137 ± 12 ^a^
	0 h fermented	1866 ± 114 ^cA^	1859 ± 62 ^bcA^	1560 ± 152 ^abB^	84 ± 8 ^b^
	24 h fermented	2144 ± 63 ^bB^	2676 ± 293 ^aA^	777 ± 115 ^dC^	36 ± 5 ^d^
	48 h fermented	2541 ± 121 ^aA^	2419 ± 225 ^aA^	1458 ± 250 ^bcB^	57 ± 10 ^c^
	72 h fermented	2354 ± 199 ^abA^	1686 ± 185 ^cB^	1745 ± 118 ^aB^	74 ± 5 ^b^
DPPH (mg TE/100 g)	Non-fermented	703 ± 2 ^cC^	1935 ± 128 ^aA^	1086 ± 59 ^aB^	155 ± 8 ^ab^
	0 h fermented	701 ± 1 ^cC^	2052 ± 231 ^aA^	1139 ± 48 ^aB^	162 ± 7 ^a^
	24 h fermented	707 ± 1 ^b^	2115 ± 73 ^a^	924 ± 144 ^b^	131 ± 20 ^c^
	48 h fermented	709 ± 2 ^bC^	2035 ± 196 ^aA^	1017 ± 145 ^abB^	144 ± 20 ^bc^
	72 h fermented	711 ± 1 ^aC^	1603 ± 200 ^bA^	1127 ± 38 ^aB^	159 ± 5 ^ab^

UD: undigested; GD: gastric digestion; ID: intestinal digestion; TPC: total phenolic content; CUPRAC: Copper (II) ion reducing antioxidant capacity; DPPH: 1,1-diphenyl-2-picrylhydrazil. Within each column, results followed by lowercase letters are significantly different (*p* < 0.05). Within each row, results followed by capital letters are significantly different (*p* < 0.05).

## Data Availability

The original contributions presented in this study are included in the article. Further inquiries can be directed to the corresponding author.
